# How did the public respond to the 2015 expert consensus public health guidance statement on workplace sedentary behaviour? A qualitative analysis

**DOI:** 10.1186/s12889-016-3974-0

**Published:** 2017-02-02

**Authors:** Benjamin Gardner, Lee Smith, Louise Mansfield

**Affiliations:** 1 Department of Psychology, Institute of Psychiatry, Psychology and Neuroscience, King’s College London, Addison House, Guy’s Campus, London, SE1 1UL UK; 2 The Cambridge Centre for Sport and Exercise Sciences, Department of Life Sciences, Anglia Ruskin University, Cambridge, UK; 3 Department of Life Sciences, College of Health and Life Sciences, Brunel University, Uxbridge, UK

**Keywords:** Sedentary behaviour, Sitting, Physical activity, Public engagement, Psychology, Qualitative, Thematic analysis

## Abstract

**Background:**

In June 2015, an expert consensus guidance statement was published recommending that office workers accumulate 2–4 h of standing and light activity daily and take regular breaks from prolonged sitting. This paper describes public responses to media coverage of the guidance, so as to understand public acceptability of the recommendations within the guidance, and perceptions of sitting and standing as health behaviours.

**Methods:**

UK news media websites that had reported on the sedentary workplace guidance statement, and permitted viewers to post comments responding to the story, were identified. 493 public comments, posted in a one-month period to one of six eligible news media websites, were thematically analysed.

**Results:**

Three themes were extracted: (1) challenges to the credibility of the sedentary workplace guidance; (2) challenges to the credibility of public health; and (3) the guidance as a spur to knowledge exchange. Challenges were made to the novelty of the guidance, the credibility of its authors, the strength of its evidence base, and its applicability to UK workplaces. Public health was commonly mistrusted and viewed as a tool for controlling the public, to serve a paternalistic agenda set by a conspiracy of stakeholders with hidden non-health interests. Knowledge exchanges focused on correcting others’ misinterpretations, raising awareness of historical or scientific context, debating current workplace health policies, and sharing experiences around sitting and standing.

**Conclusions:**

The guidance provoked exchanges of health-promoting ideas among some, thus demonstrating the potential for sitting reduction messages to be translated into everyday contexts by lay champions. However, findings also demonstrated confusion, misunderstanding and misapprehension among some respondents about the health value of sitting and standing. Predominantly unfavourable, mistrusting responses reveal significant hostility towards efforts to displace workplace sitting with standing, and towards public health science more broadly. Concerns about the credibility and purpose of public health testify to the importance of public engagement in public health guidance development.

**Electronic supplementary material:**

The online version of this article (doi:10.1186/s12889-016-3974-0) contains supplementary material, which is available to authorized users.

## Background

While the health benefits of physical activity have been recognised for many years [1, 2], an emerging literature suggests that sedentary behaviour – activity done while seated or lying down, with energy expenditure of 1.5 metabolic equivalents or less [3] – may present a health risk independent of physical activity [4–8]. Prolonged sitting time has been associated with physical and mental health conditions, such as metabolic syndrome, heart disease and depression, even when controlling for physical activity levels [4–8]. Displacing sitting with standing or light activity may mitigate detrimental health effects [9–12]. Western lifestyles are highly sedentary [13]; objective data show that, on average, US and UK adults spend approximately 60–70% of their waking hours in sedentary activity [14]. Of particular concern are office workers, who typically spend around two-thirds of the working day seated [15, 16], accumulating as much as 10.5 h of sitting per waking day [17].

Sedentary behaviour has thus emerged as a ‘new’ and prevalent behaviour of public health concern. Several national physical activity guidelines offer sedentary behaviour guidance [18–20], but recommendations have been broad, proposing that people of all ages minimise sitting time and take regular breaks from sitting. Such guidelines reflect the infancy of the evidence base, which in the early 2010s was based almost solely on observational studies [21]. Since then, smallscale exploratory intervention trials have shown encouraging associations between reducing or breaking up sitting time and health markers, such as lowered postprandial glucose excursion, oxidative stress, and fatigue [10, 11, 22, 23].

In June 2015, in response to advances within the evidence base, an expert consensus statement was published in the British Journal of Sports Medicine (BJSM), offering specific guidance for minimising sedentary office work [24]. Drawing on a quality-weighted synthesis of extant empirical research, the guidance recommends that desk-based workers aim to initially accumulate a total of 2 h/day of standing and light activity at work, progressing to 4 h/d (prorated to part-time hours [24]). It also proposes that seated work be regularly interrupted with standing work, use of sit-stand desks, or short ‘active standing’ breaks ([24] p1357). The statement was written by seven UK academics – with expertise in exercise physiology, ergonomics, sports and exercise science, and epidemiology – and the director of a national UK campaign to promote workplace standing.

The 2015 sedentary workplace guidance statement met with considerable public interest. Altmetric statistics, which collate online activities relating to a research output [25], show that (as of May 2016) the guidance has been reported on by 59 news outlets internationally [26]. Public responses to the guidance warrant close qualitative analysis, for two reasons. First, they may indicate the acceptability of its recommendations, and of workplace sitting reduction efforts more broadly. Acceptability can influence effectiveness of public health initiatives; implementation of publicly-unacceptable recommendations is unlikely to be politically or practically feasible. Acceptability of health promotion interventions among key stakeholders – for example, the willingness of desk-based office workers to comply with recommendations to stand for 2-4 h/d and take regular standing breaks – is thus central to the likelihood of improving health [27, 28]. Second, public reception of the guidance may reveal lay beliefs around sedentary behaviour, which could inform development of sedentary reduction interventions that better acknowledge such beliefs [29]. Qualitative analysis in particular permits exploration of the content of beliefs, which may serve to effectively inform sedentary reduction policy and practice [30–32]. Additionally, given that sedentary behaviour has only recently been recognised as a health concern within the scientific community [3], the general public may be largely unaware that sitting has potential health consequences over and above physical activity.

The present study used qualitative methods to describe comments from the general public posted online in response to media coverage of the 2015 sedentary office guidance statement. Our aim was to document how the guidance was received, so as to understand the acceptability of the recommendations it made, and public perceptions of sitting and standing as health behaviours.

## Methods

### Data collection

Data were posts on websites of UK news media outlets (newspapers, television broadcasters, online news) publicly made in response to a news item about the 2015 sedentary workplace guidance statement. All such news items were found to have been posted on 2nd June 2015, the publication date of the guidance. All news items were, we assume, based on a press release, which was written by the authors of the guidance [33]. All sources were searched (by LS) on 1st July 2015.

#### Newspaper websites

Current UK national newspapers were identified via Wikipedia [34], which listed ten broadsheets and twelve tabloid newspapers, all of which had public-facing websites. Given related ‘sister’ titles (e.g. The Independent, The Independent on Sunday), eleven websites were identified: dailymail.co.uk; dailystar.co.uk; express.co.uk; ft.com; theguardian.com; independent.co.uk; mirror.co.uk; morningstaronline.co.uk; thesun.co.uk; telegraph.co.uk; thetimes.co.uk. Within each website, a search function was available, into which was entered the title of the press release of the sedentary workplace guidance statement [33]. Six of the eleven websites had reported on the guidance, all six of which had allowed readers the opportunity to publicly post free-text comments, with no character limits, in response to the story, by clicking a button at the bottom of the page. All sites required respondents to log-in to registered user accounts prior to posting. There were no apparent geographical restrictions on commentators on any site, meaning that non-UK users were also able to post, though only one site publicly displayed users’ geographical information (city, country). One site (theguardian.com) allowed for posts to appear as replies to previous posts. While no comments were posted on one website, 573 comments in total were posted to the remaining five (see Table 1).

**Table 1:** Public comments posted on news media websites in response to UK media coverage of standing consensus statement

News media source	URL	No. of posts
The Express	http://www.express.co.uk/news/uk/581426/Office-workers-told-stand-for-TWO-HOURS-a-day-to-avoid-crippling-back-problems	4
The Guardian	http://www.theguardian.com/lifeandstyle/2015/jun/01/office-workers-on-feet-standing-fours-hours-day-study-health	416
The Independent	http://www.independent.co.uk/life-style/health-and-families/health-news/spending-half-the-day-on-your-feet-reduces-risk-of-heart-attacks-and-cancer-study-says-10289633.html	9
The Mail	http://www.dailymail.co.uk/news/article-3106596/Office-workers-stand-two-hours-day-Doctors-say-constant-sitting-leading-health-issues-including-obesity-cancer-Type-2-diabetes.html	126
The Mirror	http://www.mirror.co.uk/lifestyle/health/office-workers-should-stand-up-5804213	0
The Telegraph	http://www.telegraph.co.uk/news/health/news/11644683/Stand-up-for-at-least-two-hours-a-day-in-office-workers-warned.html#disqus_thread	18

#### Non-newspaper news websites

Searches were conducted of websites of five major non-newspaper UK media outlets: British Broadcasting Corporation (BBC), Independent Television (ITV), Microsoft Network (MSN), Channel 4, and Sky News. Two of these (ITV, MSN) reported on the sedentary workplace guidance, but no public responses were posted.

### Description of media reports to which posts responded

The original news reports to which the public responded covered: from the press release that accompanied the sedentary workplace guidance paper [33], the potential health impact of sitting (specific morbidities cited in five reports; premature death cited in four reports), and recommendations for 2–4 h standing (all five reports); a caveat about the dangers of prolonged static standing, from the same press release [33] (three reports; theguardian.com, mail.co.uk; telegraph.co.uk); an additional interview with one or more of the authors of the guidance, reinforcing the health risks of sitting (two reports; theguardian.com, independent.co.uk); an interview with the deputy director for health and wellbeing from Public Health England, who urged that further research be undertaken before daily targets for workplace activity are recommended (three reports; theguardian.com, independent.co.uk, express.co.uk); an interview with the Chair of the Royal College of General Physicians, who stated that employers have a responsibility to should safeguard employees’ health (one report; theguardian.com); and one interview with a representative of the Confederation of British Industry, who urged that “time spent away from the desk be balanced with the needs of the business” (one report; theguardian.com).

### Data cleaning and analysis

Each comment was extracted in full. Of the 573 comments, four were duplicates, and 76 were deemed irrelevant (e.g. responded exclusively to images used to accompany the news report) or otherwise unintelligible and so could not be coded. The final dataset thus comprised 493 eligible comments. These were analysed using Thematic Analysis, based on realist epistemological assumptions [35, 36].

Analysis followed a six-stage process [36], involving: data familiarisation; coding; theme extraction; theme review; theme naming; and narrative analysis. All three authors were involved in analysis, to confer the benefits of the multidisciplinary make-up of the team, which brought together conceptual and methodological knowledge from social and health psychology (BG), epidemiology and sports and exercise science (LS), and sociology (LM). For quality purposes and data checking, all authors independently read and reread all comments for familiarisation purposes and to note any initial points of analysis. A preliminary, inductively-derived thematic framework was constructed at a face-to-face meeting between all authors to enable a detailed coding process, and theme extraction, to be undertaken by all authors independently. Coding and theme extraction involved assigning conceptual labels to ‘events’ within the data, with multiple labels assigned to a single comment where appropriate. For analysis purposes, multiple comments from one user were treated as a single comment, to avoid over-representation of individual views. Where posts responded to previous comments, conceptual labels were assigned to a cluster of comments where the clustering was deemed meaningful and pertinent to our research question. Coded data items were collated with relevant extracts so that a review of themes could be conducted. Theme review was undertaken by BG to refine the thematic framework. This involved identifying links between conceptual labels and higher-order themes, and constantly refining the labels, themes and framework to best reflect and organise emergent insights. Naming themes, undertaken by BG, involved development of a detailed definition and analysis of theme content, and identification of concise theme labels. LM and LS inspected the final coding framework and data extracts, and verified that themes were valid and coherent representations of the data, and that theoretical saturation had been reached (Additional file 1). We intended to deal with disagreements through discussion, but no notable disagreements arose at any stage of analysis.

Quotes are provided as evidence of the validity of our analysis [37]. All quotes presented below are from different commentators. Punctuation was added to unambiguous quotes, spelling mistakes corrected, and where necessary, words added in square brackets to clarify intended meaning.

## Results

Three themes were extracted: (1) challenges to the credibility of the sedentary workplace guidance, (2) challenges to the credibility of public health, and (3) the guidance as a spur to knowledge exchange. The former two themes focused on commentators’ trust in public health science, and the latter on commentators acting as ‘citizen scientists’ by debating issues and sharing ideas inspired by the sedentary workplace guidance.

### Theme 1: Challenges to the credibility of the sedentary workplace guidance

Four aspects of the guidance were challenged: its novelty, the credibility of its authors, the strength of the evidence underpinning it, and its real-world applicability.

Many comments used humour, commonly to reflect either the perceived absurdity or perceived common-sense nature of the guidance (‘*scientists have discovered that the more birthdays you have, the longer you live’;* telegraph.co.uk). Others adopted a more serious tone to question whether the idea of reducing sitting time for health reasons was novel (“*the [Health and Safety Executive] has been recommending regular breaks from your desk for as long as I can remember”;* dailymail.co.uk).

Some comments sought to discredit the guidance authors, and the researchers involved in generating scientific evidence on workplace sitting more broadly, by questioning the legitimacy of their expertise, linking them to previous public health messages perceived to lack credibility, or questioning whether “*they practice what they preach”* (dailymail.co.uk):

*The University of Chester [affiliation of first author of the consensus statement] does not have a medical department so the authors emanating from there are not doctors.* (dailymail.co.uk)

Many comments queried the reliability of the evidence base for the guidance. Some provided real-world observations as counter-examples, while others raised concerns that researchers had misinterpreted the evidence, and offered alternative explanations of the link between sitting and health:

*We have had workers in sedentary occupations for years, yet we never saw an obese person in the 1950s and diabetes was rare. However, the graphs for the increase in sugar sales exactly match those for the increases in sugar and sucrose sales over the years. […] This has to be connected with food.* (dailymail.co.uk)

Several commentators questioned the comprehensiveness of current evidence, as they felt that the guidance neglected evidence of the negative health impact of standing, such as the risk of varicose veins.

Concerns were also raised about the real-world applicability of the guidance. Some felt its messages were contradictory and confusing:

*“Research has long linked excessive time spent sitting to increased risk of morbidity” and “standing still for prolonged periods of time also carries health risks”. OMG [oh my god].* (theguardian.com)

Many commentators highlighted settings in which they felt the guidance could not feasibly be applied, such as where employers do not permit regular breaks, or physical or social environments not conducive to standing and moving. Others felt a ‘one size fits all’ guideline for office sitting and standing was unfair. Some believed the guidance would in practice only be voluntarily adopted by those already motivated to reduce sitting time and be active, thus *“preaching to the converted”* (theguardian.com) and failing to target those who sit most. Others felt the guidance discriminated against those physically unable to stand (*“what if they are disabled or ill? This is total nonsense”*; the guardian.com), or whose occupation necessitates sitting. Several commentators sought to exempt themselves from the guidance by appealing to the nature of their occupation in this way (“*I would love to have a stand up desk, but as a transcriber, it’s impossible”;* dailymail.co.uk), or by dissociating themselves from what they perceived to be the prototypical sedentary office worker targeted by the guidance (“*I work in an office, but … I don’t sit on my butt all day”*; dailymail.co.uk). Others reported that their occupation required them to stand for longer than four hours, so felt discriminated against as they would not derive benefit from mandatory breaks from sitting.

The most common concern raised about the applicability of the guidance was that it failed to acknowledge the reality of workplace culture. While two comments attributed responsibility for workplace behaviour to individual workers, most commentators felt that individuals lacked agency over health-relevant activity within the workplace and viewed employers as solely responsible for employee health:

*[The assumption] that the choice of moving or not moving, walking, or emailing, or going to meet a co-worker instead, are matters of choice over which the employee has control – blame the victim – … is simply not true any longer in most of today’s offices.* (theguardian.com)

Many believed that health concerns fundamentally conflict with employers’ priorities, which they perceived to centre on maximising financial profit and minimising costs, even at the expense of employee health (“*wealth before health”*; dailymail.co.uk). Three comments argued that employers should – but typically do not – recognise employee health as integral to productivity (“*it’s in the interest of any employer to make sure that staff have a decent working environment … [it] reduces the number of sick days”;* theguardian.com). Many offered first-hand examples of workplace practices that they, as employees, felt were oppressive and revealed the prioritisation of productivity over employee health and wellbeing so were incompatible with taking regular breaks from sitting:

*Too frequent use of toilet facilities can get you ‘written up’ [i.e. reported to management], and/or being followed to the toilet to see what you are up to, and/or being told that you need to see a doctor to ‘correct’ this problem of frequent use.* (theguardian.com)

Several comments portrayed employers as hostile to the needs, priorities and working conditions of employees, often using slavery imagery (*“they chain you to a desk 8 hours a day”;*theguardian.com), or invoking social class conflict (“*employers demand their pound of flesh and operate a caste system”;* dailymail.co.uk). Some predicted resistance from employers towards implementing the guidance for these reasons (*“they will just lobby the government to make sure no such legislation exists”;* theguardian.com). Conversely, others felt the guidance could be used by employers to legitimise perpetuation of unfavourable working conditions (“*how about reducing working hours so people aren’t stuck at their desks for most of the day?”;* theguardian.com).

### Theme 2: Challenges to the credibility of public health

Several comments questioned the credibility of public health science more fundamentally. Public health was commonly mistrusted, and viewed as a tool for scaring the public, ultimately intended to serve a paternalistic ideological agenda rather than to truly promote evidence-based health policy and practice. Public health stakeholders were often portrayed as a homogenous outgroup (‘them’) with values antagonistic to those of the ‘real’ public (‘us’):

*Here we go again with the latest health scare. It’s amazing what they come up with to scare people.* (dailymail.co.uk)

Many commentators were suspicious of the motives of those involved in generating public health guidance, portraying public health as a conspiracy of scientists, industry, employers, politicians and the media, and public health guidance as a means of serving hidden financial interests:

*Think about it! The quoted problems did not exist ten years ago. The truth is this is yet another unnecessary fad being introduced by the medical profession. […] Good for sales of seat-less desks though! Someone is cashing in!* (dailymail.co.uk)

Others suspected that the sitting guidance was developed to allow employers to exercise control over workers, limiting personal freedom and maximising productivity while preserving the status quo of the social class system (“*bogus, management serving research. Just one more way to frighten and control the workforce”;* theguardian.com).

Some sought to discredit public health by questioning the extent to which health promotion reflects the true priorities of the general public (*“we are not all interested in exercising every frigging day”;* dailymail.co.uk), or querying the stability of public health recommendations:

*Tomorrow read how sitting for long periods is good for the heart and soul.* (dailymail.co.uk)

Some comments suggested psychological reactance, with many using humour and propositions for unhealthy behaviours to express aversion to the perceived restrictions associated with requirements for sitting or physical activity:

First post: *I leave the office for a smoke every hour or so. Takes about 300 steps of walking.* Reply: *Great! That means you only have to smoke 34 a day to pass the government target of 10,000 steps every 24 hours. Keep it up!* (theguardian.com)

### Theme 3: The guidance as a spur to knowledge exchange

Many commentators acted as ‘citizen scientists’ by sharing knowledge related to the sedentary workplace guidance, or entering into exchanges with other commentators. These comments variously sought to debate the credibility of challenges to the guidance, raise awareness of the historical, scientific or policy context of workplace sitting, and share experiences of sitting and standing, and ways to best adhere to the guidance.

Several comments were directed at earlier commentators, designed to address concerns or undermine the credibility of criticisms of the guidance:

First post: *Nonsense. […] I like sitting so I will sit.* Reply: *Of course it must be nonsense if the research says something you don’t want to hear.* (theguardian.com)

Others sought to correct misconceptions that the news item referred to physical inactivity rather than sitting, or that the health impact of sitting can be offset by physical activity:

First post: *But surely this only applies to the sedentary majority? If you run 15 miles a week I’m sure sitting down all day doesn’t matter.* Reply: *Actually, I don’t think this is true. Apparently you simply cannot run or walk enough to make up for the harm done by sitting over 8 hours a day.* (theguardian.com)

Many comments were didactic, aimed at sharing knowledge of the history of sedentary office practices, or sharing purported evidence, not included in the news item, about the mechanisms or consequences of sitting in relation to health (*“a chair which is too low in relation to the desk … can cause pain to the shoulders and neck, and give headaches’;* theguardian.com). Several comments shared propositions for alternative workplace policies more conducive to movement and health, or cited examples of more “enlightened” workplace practice from other countries (“*in Sweden they all have desks that allow them to stand as well as sit”;* theguardian.com), usually as a means of criticising workplace policy.

Many commentators endorsed the guidance by sharing personal anecdotes recounting benefits to health and wellbeing accrued from standing in the workplace, or detriments of prolonged sitting. Others offered cautionary stories of ill-health arising from standing still for long periods (“*don’t plan on standing still at a standing desk all day. That’ll hurt ya”;* theguardian.com).

Several comments offered tips for employees or employers on how to displace sitting with standing in the workplace, such as by using less comfortable seats (*“some chairs encourage you to be still and tend to ‘trap’ you in them*”; dailymail.co.uk), or, more commonly, sit-stand desks. While some endorsed commercially available options, several exchanges centred on inexpensive makeshift sit-stand desks, providing detailed specifications of what component parts to buy, and from where. Some comments offered tips for standing (“*check out the flooring first. It causes…damage to backs, legs and knees”;* dailymail.co.uk).

Other comments promoted displacing sitting with active alternatives, such as using treadmill or cycling desks, or simply moving more while seated. Some recommended taking regular breaks to engage in activity, and some endorsed physical activity within workplace spaces (e.g. stairs and corridors), though recognised that doing so could be seen by others as ‘abnormal’ behaviour:

*At least once an hour I will … run the four flights to the top of the building before sailing back down again. People look at me in a way that would suggest I’m a blithering idiot. […] Feels good.* (theguardian.com)

Several comments endorsed walking as a minimal activity required to sustain health and wellbeing (“*my brain stops unless I can walk around a bit every hour or two*”; theguardian.com), and one listed activities conducive to walking within the prototypical workplace (e.g. *“use the copier, drink water from the water fountain, go and speak to people rather than call/email them”*; theguardian.com).

While some commentators voiced concern that walking in the workplace was unfeasible because it was inaccurately seen as unproductive (“*I know someone … who was told off more than once for getting up from his desk too often even though […] it was still to do work”;* theguardian.com), others shared tips on how to take deliberately unproductive walking breaks without being reprimanded:

First post: *Someone once told me that … if we leave the room for anything, carry a paper or a notebook and you will be ignored. [On the other hand,] walk around aimlessly and someone will want to know what you are up to.* Reply: *Good idea – I will definitely try that.* (theguardian.com)

## Discussion

Public responses to an expert consensus guidance statement, which recommended that office workers accumulate 2–4 h/day of standing and light activity at work and regularly interrupt sitting with standing [24], were categorised into three themes. Responses questioned the credibility of the guidance statement, or of public health more broadly, or exchanged ideas around workplace sitting and standing, inspired by the guidance statement. These findings offer insights that may inform development of publicly acceptable sedentary behaviour reduction interventions specifically, and public health guidelines more broadly.

Public responses to the sedentary workplace guidance statement provide an insight into lay perspectives on sedentary behaviour. Some people posted comments endorsing and verifying the benefits of standing at work, and sharing tips on how to increase standing time. Others, however, demonstrated misconceptions of the health impact of sitting, with several respondents assuming that the detrimental impact of prolonged sitting could be offset by physical activity during lunchbreaks or outside of working hours. A growing literature suggests this is not the case; sedentary behaviours are associated with cardiovascular disease, cardiovascular disease risk factors, and mortality after statistical adjustment for moderate-to-vigorous intensity physical activities [5, 7, 38–40]. Some responses queried the comprehensiveness of the evidence base underpinning the guidance statement, with many suggesting that standing incurs its own health risks. Such concerns are not unfounded; prolonged standing at work increases the risk of varicose veins [41–43]. However, the dangers of static standing were acknowledged in the expert consensus statement, which noted that ‘prolonged, static, standing postures [should] be avoided’ ([24], p1360). Health risks of static standing were mentioned in three of the six media reports to which responses were posted, but this was seen as confusing by many respondents, who perceived it to contradict the message that workers should stand more and sit less. This speaks to the importance of unambiguous communication of public health messages – in this case, that sitting should be displaced not with prolonged static standing, but rather with standing and light physical activity [24]. Together, these findings demonstrate the potential for confusion, misunderstanding and misapprehension among the lay public about the health impacts of sitting and standing. Changing the behaviour of any individual requires that the individual has sufficient motivation to change [44]. Interventions to reduce sedentary behaviour should seek to increase motivation by clearly communicating the health relevance of sitting, standing and light physical activity, in language easily understood by a general public that may lack sufficient interest to process nuanced messages discerning static and ‘active’ standing [45]. Such interventions might also present evidence to tackle potential disbelief in the health value of displacing sitting with standing [24].

Many responses showed scepticism of the feasibility of implementing the recommendations of the consensus statement within the workplace. Several respondents expected that their own employers would be unwilling to adopt strategies to protect employee health if it involved purchasing expensive sit-stand workstations, or promoting standing breaks that could adversely affect worker productivity. Organisational support is important for implementation of workplace health guidelines; while, in some workplaces, individual employees may freely make personal decisions to stand and move more in line with the guidelines, effective reduction of sitting and promotion of standing and activity in the workplace will realistically depend on support from senior management [46, 47]. Managers should recognise the importance of employee health and wellbeing to business performance [47]. Engagement in physical activity can boost work performance and reduce sick leave [48]. Promoting standing in the workplace need not be financially costly; while many interventions have reduced sitting time via provision of sit-stand workstations, less costly behaviour change strategies, such as educating employees on the health impact of sitting, and encouraging self-monitoring of sitting time, have also shown promise for reducing sitting [49]. From a psychological perspective, the workplace represents an ideal setting to implement health initiatives [50]. The routinized nature of much office work increases the likelihood that health-promoting practices adopted within the workplace will become habitual, through learned associations between health actions (e.g. standing) and stable environmental cues within the workplace [51, 52]. It is of course impossible to verify our respondents’ claims about the acceptability of sitting reduction efforts within their own workplaces. Our data may rather reflect unrealistic employee perceptions of management responses to the guidance. Employee cynicism towards workplace policy arises from perceptions of senior management as lacking in integrity, competence, or trustworthiness [53]. Indeed, some comments within our dataset indicated a lack of trust in employers to protect employee health, with many viewing employers as engaging in public health efforts to serve ulterior motives such as minimising costs, and exerting greater control over employees. Effective implementation of employee health initiatives, such as campaigns to reduce sitting, will likely require taking into account the complex, interlinked systems of employees’ needs, working cultures, and organisational structures [53].

We documented instances of acceptance and of rejection of the sedentary workplace guidance. Some respondents appeared acceptant of the potential health benefits of reducing sitting and increasing standing, and posted messages to engage in an exchange of ideas on how to implement the guidance. This demonstrates the potential for public health guidance to be championed by ‘citizen public health scientists’, who reconstruct and translate it into everyday social contexts [54]. In contrast, however, many other respondents challenged the guidance, some using examples of apparently non-credible previous health messages to discredit the sedentary behaviour statement. This demonstrates the broader, historical context in which the public considers public health communications. UK public health campaigns may be disadvantaged due to well-documented British public health controversies that persist in the public consciousness [55]. For example, doubts were raised over the safety of the combined measles, mumps and rubella (MMR) vaccine following a study, since retracted and discredited, that linked MMR vaccination with autism. Public unease persisted when the then-chief medical officer refused to sanction use of single vaccines [55], and the British Prime Minister refused to publicly state whether his son had received the MMR vaccine. Additionally, in 1996 the UK government announced a link between bovine spongiform encephalopathy (BSE) and variant Creutzfeld Jakobs Disease, despite repeated prior public statements that there was no link and that beef consumption was harmless. The BSE case in particular has been credited for leading to a ‘collapse’ in public trust in government-sponsored public health messages [55, 56], with suspicions raised that government colluded with industry to cover up a known health threat [57].

Mistrust presents a considerable challenge to public acceptance of public health messages. People cannot be expected to freely adhere to public health guidance on limiting sitting time where they do not view sitting time to be harmful, or lack confidence in the effectiveness of breaking up sitting for reducing such health risks [58]. The mistrust observed within our dataset may at least partly be attributable to a lack of public engagement in the generation of the sedentary behaviour guidance statement, which was developed by academic experts alone [24]. This approach to guidance development separates researchers and public health practitioners from the public [31]. Some of the adverse responses that we documented, such as those reaffirming a commitment to sitting (e.g., *‘I like sitting so I will sit’*), may represent attempts to resist influence from an outgroup (*i.e.*, ‘academic experts’), to which the public believes it neither belongs nor shares its interests, priorities, or experiences. Other responses, by proposing counter-examples that contradicted the central message to sit less in the workplace, implicitly questioned the evidence synthesis process used to generate the sedentary behaviour consensus statement. Such concerns demonstrate the need for public involvement in the development of public health guidance, and greater transparency of the processes of identifying and synthesising the evidence base informing such guidance. Public involvement in guidance development allows for the integration of lay knowledge arising from lived experience [31], and may achieve greater acceptance among those most directly affected by such guidance. Public involvement is standard practice among many public health bodies, such as the National Institute for Health and Care Excellence [59].

### Limitations

Limitations of our study must be acknowledged. Our dataset comprises comments posted to news media websites, yet there are multiple online media through which the public can respond to public health messages, such as Twitter. We excluded tweets from analysis because the 140 character limit imposed by Twitter limits the richness of data, unlike limitless free-text entry forms on news websites. We excluded posts from other social media sites due to the resource-intensity of reliably and systematically identifying and synthesising all such posts. We do not, however, expect that views posted to news websites should systematically differ in their thematic content to those on other media.

It is unclear how representative the views of those who posted comments to news websites are of the general public. One US survey of over 2,000 adults found that online news consumers were likely to be younger, more educated, and have a higher income than others [60]. The same survey also reported that only 25% of respondents had ever posted online comments about news stories [60]. Additionally, people who actively contribute to discourse around workplace sedentary behaviour, or public health more broadly, may perhaps have greater interest in or more pronounced views on these topics than does the general public. For these reasons, we cannot establish whether the strength or content of views documented here are representative of the general public. Nonetheless, our data reveal important concerns that may act as barriers to acceptability of sitting reduction initiatives, and of public health communications more broadly, among the general public.

While we treated our data as responses to the sedentary workplace guidance statement, responses were made to secondary, media reports of the statement. While all five news reports that elicited public responses covered the two main recommendations of the guidance statement, some also reported the views of other stakeholders, such as a British industry spokesperson (one report), and a public health specialist not involved in developing the guidance (three reports). These additional views may have influenced public responses. However, while post-peer-review websites allow the public to post comments on scientific outputs, we deemed it unlikely that the lay public would engage with such sites. Additionally, many public health messages are effectively disseminated to the public via secondary media reports. We thus believe our data to represent valid and insightful responses to a public health guidance statement disseminated to them via regular channels.

## Conclusion

Public responses to the 2015 expert consensus sedentary workplace guidance statement reveal important potential barriers to public acceptability of sitting time reduction in the workplace, and of public health more broadly. Sedentary behaviour reduction interventions should seek to discern and communicate clearly the evidence basis for the health impacts of sitting, standing, and physical activity. Greater public engagement in public health guidance development may perhaps assuage the challenges we documented to the credibility and motives of public health science and its stakeholders.

## Additional files

Additional file 1: Final coding tree. (DOCX 17 kb)

## Abbreviations

BBC: British Broadcasting Corporation
BSE: Bovine spongiform encephalopathy
ITV: Independent Television
MMR: Measles, mumps and rubella
MSN: Microsoft Network

## References

[1] Morris JN, Heady JA, Raffle PA, Roberts CG, Parks JW (1953). Coronary heart-disease and physical activity of work. Lancet.

[2] Warburton DE, Nicol CW, Bredin SS (2006). Health benefits of physical activity: the evidence. CMAJ.

[3] Sedentary Behaviour Research Network (2012). Standardized use of the terms “sedentary” and “sedentary behaviours”. Apply Physiol Nutr Metab.

[4] Bertrais S, Beyem-Ondoua JP, Czerinchow S, Galan P, Hercberg S, Oppert JM (2005). Sedentary behaviors, physical activity, and metabolic syndrome in middle-aged French subjects. Obes Res.

[5] Thorp AA, Owen N, Neuhaus M, Dunstan DW (2011). Sedentary behaviors and subsequent health outcomes in adults a systematic review of longitudinal studies, 1996–2011. Am J Prev Med.

[6] Teychenne M, Ball K, Salmon J (2010). Physical activity, sedentary behavior and depression among disadvantaged women. Health Educ Res.

[7] Grontved A, Hu FB (2011). Television viewing and risk of type 2 diabetes, cardiovascular disease, and all-cause mortality: a meta-analysis. JAMA.

[8] Healy GN, Matthews CE, Dunstan DW, Winkler EA, Owen N (2011). Sedentary time and cardio-metabolic biomarkers in US adults: NHANES 2003–06. Eur Heart J.

[9] Alkhajah TA, Reeves MM, Eakin EG, Winkler EA, Owen N, Healy GN (2012). Sit-stand workstations: a pilot intervention to reduce office sitting time. Am J Prev Med.

[10] Buckley JP, Mellor DD, Morris M, Joseph F (2014). Standing-based office work shows encouraging signs of attenuating post-prandial glycaemic excursion. Occup Environ Med.

[11] Dunstan DW, Kingwell BA, Larsen R, Healy GN, Cerin E, Hamilton MT, Shaw JE, Bertovix DA, Zimmet PZ, Salmon J, Owen N (2012). Breaking up prolonged sitting reduces postprandial glucose and insulin responses. Diabetes Care.

[12] Healy GN, Dunstan DW, Salmon J, Cerin E, Shaw JE, Zimmet PZ, Owen N (2008). Breaks in sedentary time: Beneficial association with metabolic risk. Diabetes Care.

[13] Bennie JA, Chau JY, van der Ploeg HP, Stamatakis E, Do A, Bauman A (2013). The prevalence and correlates of sitting in European adults - a comparison of 32 Eurobarometer-participating countries. Int J Behav Nutr Phys Act.

[14] Stamatakis E, Hamer M, Tilling K, Lawlor DA (2012). Sedentary time in relation to cardio-metabolic risk factors: differential associations for self-report vs accelerometry in working age adults. Int J Epidemiol.

[15] Ryde GC, Brown HE, Gilson ND, Brown WJ (2014). Are we chained to our desks? Describing desk-based sitting using a novel measure of occupational sitting. J Phys Act Health.

[16] Ryan CG, Dall PM, Granat MH, Grant PM (2011). Sitting patterns at work: objective measurement of adherence to current recommendations. Ergonomics.

[17] Smith L, Hamer M, Ucci M, Marmot A, Gardner B, Sawyer A, Wardle J, Fisher A (2015). Weekday and weekend patterns of objectively measured sitting, standing, and stepping in a sample of office-based workers: the Active Buildings study. BMC Pub Health.

[18] Australian Government Department of Health (2014). Make your move - sit less. Be active for life!.

[19] UK Department of Health (2011). Start active, stay active: A report on physical activity for health from the four home countries’ chief medical officers.

[20] Tremblay MS, Leblanc AG, Janssen I, Kho ME, Hicks A, Murumets K, Colley RC, Duggan M (2011). Canadian sedentary behaviour guidelines for children and youth. Appl Physiol Nutr Metab.

[21] Brown WJ, Bauman AE, Bull FC, Burton NW (2012). Development of evidence-based physical activity recommendations for adults (18–64 years).

[22] Takahashi M, Miyashita M, Park J-H, Sakamoto S, Suzuki K (2015). Effects of breaking sitting by standing and acute exercise on postprandial oxidative stress. Asian J Sports Med.

[23] Wennberg P, Boraxbekk C-J, Wheeler M, Howard B, Dempsey PC, Lambert G, Eikelis N, Larsen R, Sethi P, Occleston J, Hernestal-Boman J, Ellis KA, Owen N, Dunstan DW (2016). Acute effects of breaking up prolonged sitting on fatigue and cognition: a pilot study. BMJ Open.

[24] Buckley JP, Hedge A, Yates T, Copeland RJ, Loosemore M, Hamer M, Bradley G, Dunstan DW (2015). The sedentary office: an expert statement on the growing case for change towards better health and productivity. Brit J Sports Med.

[25] Altmetric. www.altmetric.com. Accessed 23 May 2016.

[26] Altmetric. The sedentary office: an expert statement on the growing case for change towards better health and productivity. Overview of attention for article published in British Journal of Sports Medicine, June 2015. https://www.altmetric.com/details/4079139. Accessed 30 June 2015. 10.1136/bjsports-2015-094618 26034192

[27] Craig P, Dieppe P, Macintyre S, Michie S, Nazareth I, Petticrew M (2008). Developing and evaluating complex interventions: the new Medical Research Council guidance. BMJ.

[28] Michie S, Atkins L, West R (2014). The behaviour change wheel: a guide to designing interventions.

[29] Gardner B, Davies A, McAteer J, Michie S (2010). Beliefs underlying UK parents’ views towards MMR promotion interventions: a qualitative study. Psychol Health Med.

[30] Beresford P (2002). User involvement in research and evaluation: Liberation or regulation?. Soc Policy Society.

[31] Israel BA, Schulz AJ, Parker EA, Becker AB (1998). Review of community-based research: assessing partnership approaches to improve public health. Annu Rev Public Health.

[32] Newman J, Barnes M, Sullivan H, Knops A (2004). Public participation and collaborative governance. J Soc Policy.

[33] British Journal of Sports Medicine (2015). Get up and stand up for at least 2 hours daily during working hours, office workers advised.

[34] Wikipedia. List of newspapers in the United Kingdom. https://en.wikipedia.org/wiki/List_of_newspapers_in_the_United_Kingdom. Accessed 23 May 2016.

[35] Braun V, Clarke V (2006). Using thematic analysis in psychology. Qual Res Psychol.

[36] Clarke V, Braun V (2013). Teaching thematic analysis: Overcoming challenges and developing strategies for effective learning. The Psychologist.

[37] Mays N, Pope C (1995). Rigour and qualitative research. BMJ.

[38] Proper KI, Singh AS, van Mechelen W, Chinapaw MJ (2011). Sedentary behaviors and health outcomes among adults: a systematic review of prospective studies. Am J Prev Med.

[39] Smith L, Thomas EL, Bell JD, Hamer M (2014). The association between objectively measured sitting and standing with body composition: a pilot study using MRI. BMJ Open.

[40] van der Ploeg HP, Chey T, Korda R (2012). Sitting time and all-cause mortality risk in 222,497 Australian adults. Arch Intern Med.

[41] Kontosić I, Vukelić M, Drescik I, Mesaros-Kanjski E, Materljan E, Jonjić A (2000). Work conditions as risk factors for varicose veins of the lower extremities in certain professions of the working population of Rijeka. Acta Med Okayama.

[42] Laurikka JO, Sisto T, Tarkka MR, Auvinen O, Hakama M (2002). Risk indicators for varicose veins in forty- to sixty-year-olds in the Tampere varicose vein study. World J Surg.

[43] Tüchsen F, Hannerz H, Burr H, Krause H (2005). Prolonged standing at work and hospitalisation due to varicose veins: a 12 year prospective study of the Danish population. Occup Environ Med.

[44] Michie S, van Stralen MM, West R (2011). The behaviour change wheel: a new method for characterising and designing behaviour change interventions. Implement Sci.

[45] Petty RE, Cacioppo JT (1986). The elaboration likelihood model of persuasion. Communication and persuasion: central and peripheral routes to attitude change.

[46] Healy GN, Eakin EG, Lamontagne AD, Owen N, Winkler EA, Wiesner G, Gunning L, Neuhaus M, Lawleer S, Fjeldsoe BS, Dunstan DW (2013). Reducing sitting time in office workers: short-term efficacy of a multi-component intervention. Prev Med.

[47] Pronk NP, Kottke TE (2009). Physical activity promotion as a strategic corporate priority to improve worker health and business performance. Prev Med.

[48] Proper K, van Mechelen W. Effectiveness and economic impact of worksite interventions to promote physical activity and healthy diet. Geneva: World Health Organization; 2008.

[49] Gardner B, Smith L, Lorencatto F, Hamer M, Biddle SJH (2016). How to reduce sitting time? A review of behaviour change strategies used in sedentary behaviour reduction interventions among adults. Health Psychol Rev.

[50] Stokols D, Pelletier KR, Fielding JE (1996). The ecology of work and health: research and policy directions for the promotion of employee health. Health Educ Quart.

[51] Lally P, Gardner B (2013). Promoting habit formation. Health Psychol Rev.

[52] Lally P, Wardle J, Gardner B (2011). Experiences of habit formation: a qualitative study. Psychol Health Med.

[53] Albrecht SL (2002). Perceptions of integrity, competence and trust in senior management as determinants of cynicism towards change. Pub Admin Manage.

[54] Irwin A, Wynne B (1996). Misunderstanding science? The public reconstruction of science and technology.

[55] Bellaby P (2003). Communication and miscommunication of risk: understanding UK parents’ attitudes to combined MMR vaccination. BMJ.

[56] Gerodimos R (2004). The UK BSE Crisis as a Failure of Government. Pub Admin.

[57] Bartlett DMC (1998). Mad cows and democratic governance: BSE and the construction of a ‘free market’ in the UK. Crime Law Soc Chang.

[58] Floyd DL, Prentice-Dunn S, Rogers RW (2000). A meta-analysis of research on protection motivation theory. J Appl Soc Psychol.

[59] National Institute for Health and Care Excellence. Public involvement. https://www.nice.org.uk/about/nice-communities/public-involvement. Accessed 19 May 2016.

[60] Purcell K, Rainie L, Mitchell A, Rosenstiel T, Olmstead K. Understanding the participatory news consumer: How internet and cell phone users have turned news into a social experience. http://www.journalism.org/files/legacy/Participatory_News_Consumer.pdf. Accessed 5 August 2016.

