# Supplementary material for: How did the public respond to the 2015 expert consensus public health guidance statement on workplace sedentary behaviour? A qualitative analysis
Source: BMC Public Health. 2017 Feb 2;17:47. doi: 10.1186/s12889-016-3974-0 (PMC5288998; doi:10.1186/s12889-016-3974-0)
Supplement: Additional file 1: — Final coding tree. (DOCX 17 kb) [file 12889_2016_3974_MOESM1_ESM.docx]

**Additional file 1. Final coding tree**

**Theme 1: Challenges to credibility of the sedentary workplace guidance**

1. Challenges to novelty of guidance
   1. Guidance as common-sense
   2. Guidance as not new
2. Challenges to credibility of authors
   1. Authors lack real-world expertise
   2. Authors’ credibility tainted by previous public health messages
   3. Authors’ credibility undermined by assumed failure to adhere
3. Challenges to evidence base for guidance
   1. Challenge to credibility of scientific evidence
      1. Counter-arguments stemming from common-sense/personal beliefs
      2. Counter-arguments stemming from real-world observations
      3. Evidence-base for guidance seen to be incomplete/unstable
      4. Apparent inconsistencies/contradictions/confusion in evidence base
4. Challenges to real-world applicability of guidance
   1. Unrealistic
      1. Fails to apply to all people equally (‘one size does not fit all’)
         1. Fails to apply to:
            1. those physically unable to stand
      2. Fails to map on to specific contexts
         1. Exempt settings/roles
            1. those who job requires sitting
            2. those whose job does NOT involve sitting (so already standing)
         2. Would only be adopted by a few (those already motivated to protect health; ‘preaching to the converted’)
      3. Fails to apply to real-world workplace culture
         1. Employees or employers responsible for workplace behaviour?
         2. Conflict with employer priorities
            1. Profit over employee health
            2. Costs over employee health
            3. Productivity over employee health
         3. Controlling and oppressive workplaces/employers
            1. Employee concern around how guidance used by employers

**Theme 2: Challenges to credibility of public health**

1. PH as outgroup
   1. ‘Them’ vs ‘us’
2. Trust/mistrust of purpose and motives of PH/stakeholders
   1. Perceived conspiracy between PH stakeholders
   2. Perceived purpose of PH
      1. to control/scare the public
      2. is to maintain and serve hidden interests
         1. financial gain for stakeholders
         2. productivity for employers
      3. financial gain for PH stakeholders
3. PH guidance in general lacks real-world applicability
   1. Places more priority on health than does the general public
   2. Faddish
4. Psychological reactance
   1. Humour
   2. Behaviour compensation

**Theme 3: Guidance as spur to knowledge exchange**

1. Debate/dialogue between readers
   1. Correcting misconceptions about guideline
   2. Correcting perceived inaccuracies in other posters’ interpretation of evidence
2. Information about historical context
3. Information about scientific context
   1. Sharing knowledge of scientific evidence base
   2. Adding new information to the evidence base
      1. Examples of successful ‘standers’
4. Activism and calls to arms for policy change
   1. Examples of good practice from other countries/settings
5. Personal experiences of standing
   1. Pro-guidance
      1. Health benefits of standing
      2. Negative experiences of sitting
      3. Enjoyment
   2. Anti-guidance
      1. Health harms of standing
6. Ways to offset sitting
   1. Effective ways to stand
      1. Modify or change chair/seat
      2. Sit-stand desks
         1. Commercial desks
         2. Makeshift standing desks
      3. Best standing practice
   2. Replace sitting with PA
      1. At-desk: Treadmill desks or equivalent
      2. Away from desk: Replace sitting with mod/vig PA within workplace
         1. Responses from others to workplace PA
   3. Increase physical activity elsewhere (a misconception)
      1. Disbelief that PA cannot offset sitting
      2. Correcting PA misconception
   4. Replace sitting with walking/moving
      1. Identify movement-conducive work activities
